# Near Their Thresholds for Detection, Shapes Are Discriminated by the Angular Separation of Their Corners

**DOI:** 10.1371/journal.pone.0066015

**Published:** 2013-05-31

**Authors:** J. Edwin Dickinson, Jason Bell, David R. Badcock

**Affiliations:** 1 School of Psychology, The University of Western Australia, Perth, Western Australia, Australia; 2 Research School of Psychology, The Australian National University, Canberra, Australian Capital Territory, Australia; University of Leuven, Belgium

## Abstract

Observers make sense of scenes by parsing images on the retina into meaningful objects. This ability is retained for line drawings, demonstrating that critical information is concentrated at object boundaries. Information theoretic studies argue for further concentration at points of maximum curvature, or corners, on such boundaries [1]–[3] suggesting that the relative positions of such corners might be important in defining shape. In this study we use patterns subtly deformed from circular, by a sinusoidal modulation of radius, in order to measure threshold sensitivity to shape change. By examining the ability of observers to discriminate between patterns of different frequency and/or number of cycles of modulation in a 2x2 forced choice task we were able to show, psychophysically, that difference in a single cue, the periodicity of the corners (specifically the polar angle between two points of maximum curvature) was sufficient to allow discrimination of two patterns near their thresholds for detection. We conclude that patterns could be considered as labelled for this measure. These results suggest that a small number of such labels might be sufficient to identify an object.

## Introduction

A central task of the visual system is to segment objects from the background of a scene and to identify them so that appropriate actions can be planned. Several approaches could facilitate the ability to recognise and discriminate between shapes. The visual system could identify critical local properties and develop a parametric description of those local features, using variation of those parameters to discriminate between shapes and detect when collections of those features are present in order to recognise previously learnt shapes. A second alternative is to use a more rigid set of generic shape templates and to monitor the “goodness of fit” of the set of generic shapes to object contours to provide a parametric representation. Discrimination would then be supported by changes in activation across the family of generic shape templates and recognition could be supported by particular patterns matching previously learnt object contours. A third alternative is to have very specific templates for particular objects such that activation of the template identifies the object. This latter possibility has been discussed at length elsewhere [4], [5] and has been rejected as a general solution because of the very substantial number of templates that would be required to account for size, viewpoint and orientation invariances for every recognised object.

However, there is data supporting both of the first two alternatives. Cadieu et al. [6] present a model based on the earlier physiological recordings of Pasupathy & Connor [7], [8] showing neurones in V4 that respond selectively to curved features at specific polar angles relative to the centre of an object. Cadieu et al. [6] provided an account that detects collections of such features for object recognition regardless of the position of the collection within the visual field. Such a model would provide a graded response when local features varied and optimal response when all of a particular set was present.

**Figure 1:**
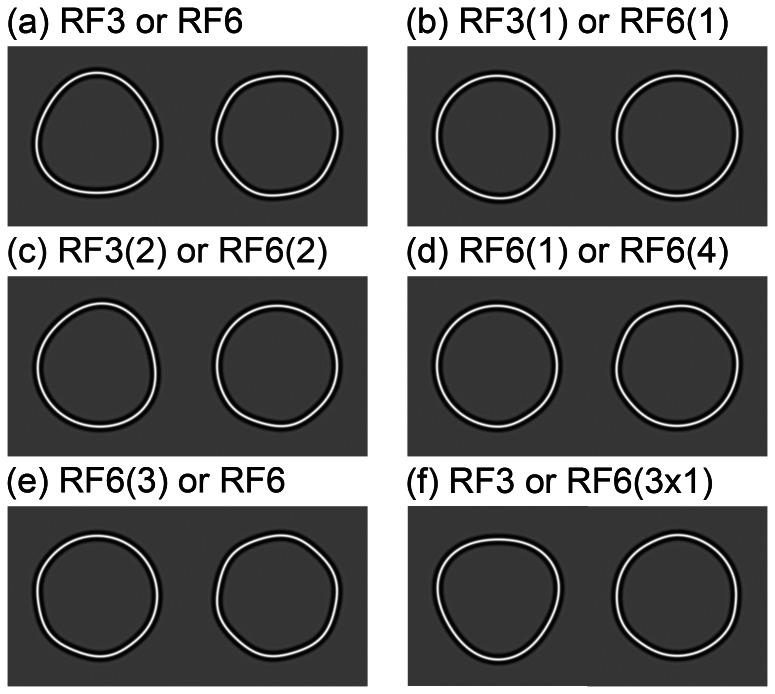
Example stimulus pairs. The pairs of test stimuli are those used in the 2x2FC tasks. One of the two stimuli was presented in each trial of a block. Each trial had two intervals, with the test stimulus presented in one interval and a circular reference stimulus presented in the other. The observer was required to identify the interval in which the test stimulus was presented (detection) and then identify which of the pair of test stimuli was presented in a second response (identification). The amplitudes, *A*, of the stimuli shown are 0.05 and 0.0135 for the patterns containing RF3 and RF6. A pattern with an amplitude of greater than 1/(1+ω^2^), where ω is the frequency of the modulating sine function, has points of maximum concave curvature in addition to the points of maximum convex curvature. None of the patterns used in the experiments had amplitudes this large. The examples shown have half of this amplitude. The pairs are: (a) complete RF3 and RF6 patterns, patterns deformed from circular by a sinusoidal modulation of radius, with a frequency of 3 and 6 cycles of modulation in 2π radians respectively; (b) an RF3 and an RF6 pattern with a single, (1), cycle of modulation each; (c) an RF3 and an RF6 pattern with two, (2), consecutive cycles of modulation; (d) an RF6 pattern with one (1) cycle of modulation and an RF6 pattern with four (4) consecutive cycles of modulation; (e) an RF6 pattern with three (3) cycles of modulation and a complete RF6 pattern; (f) a complete RF3 pattern and an RF6 pattern with 3 alternate single cycles of modulation (3x1).

The second alternative is explicitly represented by the model of Poirier and Wilson [9] where contours are decomposed into a set of responses in a range of detectors. The detectors are modelled as radial frequency (RF) selective. A radial frequency pattern is created by modulating the radius of a circle by a sinusoid as a function of polar angle. This creates a contour that deforms outwards from the circle when the sinusoid increases in value and inwards when it decreases (see Fig 1). Different frequencies of sinusoidal modulation can produce different shaped contours (e.g. 3 cycles can approximate a triangle, 4 a square, 5 a pentagon). The model produces a value for each radial frequency indicating the amount of modulation that would be required to produce the contour when all components are combined. This would imply a set of underlying generic templates if the processing of one radial frequency in a combined contour could be seen to be independent of the processing of a different radial frequency. There is psychophysical data arising from masking and subthreshold summation techniques which are consistent with this interpretation [10] but in those cases observers were not required to identify the shape. Instead, they only needed to discriminate one pattern from another. With the smallest amounts of detectable modulation it is likely only the most sensitive channel for the presented shape would be active. Independent templates, or channels, would imply that as soon as the shape channel had sufficient signal to be active observers should be able to know the identity of the shape driving that channel; assuming such channels act as labelled lines. It is, at present, unclear whether that is the case.

In the experiments that follow, a set of patterns have been chosen to examine the ability to distinguish between different radial frequencies at the minimum detectable modulation level. Conditions distinguish between differences in radial frequency (different shapes) and differences in the number of lobes of deformation (completeness of RF pattern). We also examine the minimum criteria for recognition by considering whether single lobe features can be distinguished as readily as those with repeated lobes.

At threshold, performance in discrimination of patterns with a single cycle of modulation has the potential to determine whether a continuous or rectified measure of curvature with respect to circular is used in the analysis of shape and, therefore, whether shape might be decomposed into radial frequency components. Rectification could be considered explicit in the model of Poirier and Wilson [9], where only curvature that is convex with respect to circular is used in the shape analysis. The frequency spectrum of the curvature of a pattern with one complete cycle of modulation would be peaked at the frequency of modulation if a continuous measure were used but not if a rectified measure were employed. Single lobes of different radial frequencies would also differ in the polar angle subtended by their respective curvature features and thus address whether suggestions that polar angles are a critical element of the shape code should refer to the extent of a single lobe or, instead, the angle between repeated points of maximum curvature, or corners.

Prior experiments have shown that modulation thresholds for shape discrimination do improve with multiple lobes more than can be accounted for by independent detection of the individual lobes [11], [12]; a marker of global integration of information around the contour. However, it is important to distinguish between the use of different underlying global processes such as generic templates and the simple counting of the number of lobes as that number also varies with changing radial frequency.

To address this issue radial frequency patterns are employed that have the same radial frequency but different numbers of cycles. Again observers will be asked to discriminate the modulated pattern from a circle and to then indicate which of two patterns was presented. If the number of lobes is detectable at threshold then the patterns should be discriminable. However, the patterns should maximally stimulate the same RF detectors (labelled lines) and therefore should not be discriminable when matched for salience by working at threshold signal level if activation of labelled lines is the critical code.

Thus by using weak signals and measuring the amount of modulation required to distinguish the chosen RF patterns from circles, it is possible to determine whether observers are able to identify the radial frequency of a pattern at the same modulation level. If they can it will be an indication that labelled lines are contributing to performance.

Our experiments are, therefore, analogous to those previously used to demonstrate the existence of information channels sensitive to different spatial frequencies using a 2x2 forced choice procedure [13], [14]. In those experiments observers could identify which of two sinusoidal gratings (sufficiently different in spatial frequency) presented near their contrast detection threshold was present, in any particular trial, as reliably as they could report whether they were there at all, or in which interval of two they appeared. This has been interpreted as evidence that not only do individual neurons respond to a narrow range of spatial frequency, they project to other neurons in a way that retains the information of their preferred spatial frequency, i.e. labelled information lines [28]. Identification of a particular stimulus under this scheme only requires that the magnitude of the stimulus reaches its threshold for detection. By extension, discrimination between two stimuli detected within different labelled lines is possible at their threshold for detection [15].

For this study orientation of the test pattern was randomized across trials, to prevent the monitoring of a single local segment of the contours, and observers were required to detect the deformation in the test pattern and identify it as one particular pattern of a pair. The tests were binary in the sense that if the two patterns could be discriminated at their thresholds for detection they were assumed to be detected by discrete labelled information lines. We used RF3 and RF6 patterns as they differ in frequency by one octave and therefore it is possible to create a stimulus with alternate cycles of an RF6 pattern removed (a separation of 2π/3 radians between points of peak convex curvature). The separation of points of maximum convex curvature would then have the same polar angle between points of peak curvature in an RF3 pattern (see Figure 1(f)).

Figure 1 depicts the conditions employed, which were designed to test whether perfect discrimination was possible for (a) complete RF patterns with different frequencies, RF3 or RF6; (b) patterns with different frequencies but only a single cycle of modulation, RF3(1) or RF6(1); (c) patterns with different frequencies and two continuous cycles of modulation, RF3(2) or RF6(2); (d) and (e) patterns with the same frequency but different numbers of continuous cycles of modulation, RF6(1) or RF6(4) and RF6(3) or RF6; and (f) patterns with different frequencies of modulation but the same spatial arrangement of points of maximum convex curvature, RF3 or RF6(3x1). The two patterns in condition (f) were an RF3 pattern and an RF6 pattern on which alternate cycles of modulation had been removed. As mentioned earlier the RF6 pattern modified in this way has the same periodicity of points of maximum convex curvature as an RF3 pattern.

Mechanisms for the detection of RF patterns are narrowly tuned for radial frequency [22] and so we might expect the complete RF patterns shown in Pair 1(a) to be discriminated at their thresholds for detection. We would expect the single cycle pair shown in Figure 1(b) to be perfectly discriminated if a continuous measure of curvature were employed and patterns are decomposed into radial frequency components. If, however, shapes are identified on the basis of the periodicity of adjacent points of maximum convex curvature, then they should not be perfectly discriminated because their frequency spectra would increase towards, and peak at, zero (there is no repetition of curvature features and therefore no periodicity of signal). In contrast, the frequency spectra of the patterns containing two cycles of modulation (Pair 1(c)) would peak an octave apart and thus would be predicted to be discriminable at their threshold for detection for either decomposition scenario. Pairs 1(d) and 1(e) differ only in number of cycles of modulation of a given frequency, thus decomposition into radial frequency components or representation by the periodicity of points of maximum convex curvature would render them not discriminable at threshold (eliminating the possibility of explicit templates with different numbers of cycles of modulation facilitating discrimination, consistent with Schmidtmann, Kennedy, Orbach & Loffler’s argument [16] that all of the template must be present to allow global integration of signal). In the case of Pair 1(f), if discrimination at threshold is primarily made on the periodicity of points of maximum convex curvature then we can expect an excess of false positive identifications of an RF3 pattern because both frequency spectra are peaked at a frequency of three cycles per 2π radians. This excess can be detected by an artificial decrease in the threshold for identification. A compensating deficit in detections of distributed RF6 modulation would result in an increase in its threshold for identification.

## Materials and Methods

### Stimuli

The stimuli were derived from the radial frequency (RF) patterns [17] used by Poirier and Wilson [9], paths deformed from circular by a sinusoidal modulation of the radius. RF pattern radius is given by
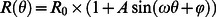
(1)where *θ* is the angle made with the x axis, *R*_0_ the mean radius (1° in all patterns), *A* the amplitude of modulation (expressed as a proportion of the mean radius, R_0_), *ω* the frequency of modulation (cycles in 2π radians), and *φ* the phase of the sinusoidal modulator. Phase was randomized in each pattern. The mean radius *R*_0_ of all patterns was 1°. In order to reduce the number of cycles on the RF patterns we used closed contours where the modulation of the radius was restricted to a limited range of θ. To ensure a smooth transition from the modulated to the circular sectors of the partially modulated patterns a D1 (first derivative of a Gaussian), matching the amplitude and maximum slope of the particular sine wave modulator, was used to replace the half cycles at the two ends of the train of cycles of modulation, following the procedure employed by Loffler et al. [11]. The modulation of patterns with only one cycle of modulation therefore conformed solely to the appropriate amplitude profile of the first derivative of a Gaussian, with the period of the single cycle proportional to the standard deviation of the Gaussian. The paths had a D4 (fourth derivative of a Gaussian) luminance profile in cross-section with a frequency spectrum peaking at 8 c/deg also following Loffler et al. [11] and, earlier, Wilkinson et al. [17].

The angle the path makes with a circle as a function of θ is given by
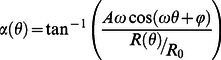
(2)which for the small amplitudes of modulation in radius employed in this study is approximately sinusoidal. The rate of change of this angle with respect to circular, which is the quantity adopted by Poirier and Wilson to represent curvature, is also approximately sinusoidal.

### Observers

Four observers participated in the study. JB and ED are authors. RO and VB are experienced psychophysical observers but were naïve to the purpose of the study. All observers had normal visual acuity. ED has a divergent squint and observed the stimuli monocularly using an eye patch. Although all of the observers were experienced psychophysical observers they were trained in neither the observation of these particular stimuli nor in this particular experimental paradigm. We would therefore expect to obtain the same results from observers that were unfamiliar with psychophysical experiments. The data might, however, be expected to be more variable.

### Ethics Statement

All experiments of this study were approved by the University of Western Australia ethics committee and therefore were conducted in accordance with the Declaration of Helsinki. Both of the naïve observers in this study were satisfied that they were given sufficient information regarding the experiment to give informed consent to their participation. Written evidence of that consent was obtained.

### Apparatus

Stimuli were created using custom software in MatLab 5.3 (Mathworks, Natick, MA, USA) and drawn to the display from the frame buffer of a Cambridge Research Systems VSG 2/4 graphics card housed in a PC (Pentium II, 400MHz). The observers viewed a Hitachi Accuvue 4821 monitor (100 Hz refresh rate) from a distance of 132 cm, stabilized with a chin rest. Each square pixel of a 752×752 display subtended 1’ of arc at the observing distance. Observers were informed that the stimuli would be presented at the centre of the screen but the centre of each pattern was allowed to vary ± 7’ of visual angle from the centre of the screen on the vertical and horizontal axes at random to render the edges of the screen unhelpful as positional references for analysis of shape. No fixation point was used. Luminance calibration was performed using a Cambridge Research Systems OPTICAL OP 200-E photometer (Head model number 265) and associated software. Background luminance was 45 cd/m^2^. The maximum Weber contrast of the stimuli was 1.

### Procedure

A two by two forced choice (2x2FC) experimental paradigm was employed [15] where the observer was required to make two responses to a pair of stimuli presented in sequence and separated in time by a 300 ms inter-stimulus interval. Each stimulus was displayed for 160 ms, a stimulus duration similar to that used in other studies that have demonstrated integration of shape information in RF patterns [11], [12], [18]. One interval contained a reference stimulus, a circular pattern (*A* = 0 in Eq. 1), while the other contained a modulated test stimulus of one of two possible types for each condition of the experiment. The order of presentation of the reference and test stimuli was randomized. The first response of the observer was to report which of the two intervals contained the test stimulus (detection) and the second, to report which of the two alternative test patterns was presented (identification). Each block of trials only contained stimuli of one particular condition and the observer was shown supra-threshold examples of the stimuli prior to each block. No feedback was provided. The method of constant stimuli (MOCS) was used to measure the probability of correct response in the two tasks over a range of 7 amplitudes of modulation for each of the two types of test stimuli. Sixty trials were performed at each amplitude value over three separate blocks of 140 trials. The data of the four observers were similar and were, therefore, combined. A Quick function [19], [20] was fitted to each of the four combined sets of psychometric data (two representing the probabilities of detection of the two types of test stimuli versus amplitude of deformation, and two describing the probability of each type being reported as present, versus amplitude of modulation). The Quick function has the form
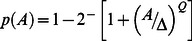
(3)where *p* is the probability of correct response, *A* is the modulation amplitude as a proportion of the radius of the unmodulated circle, *Δ* is the threshold at the 75% correct performance level and *Q* is a measure of the slope of the psychometric function. If the probabilities of detection and identification of each pattern of a pair are equal at all modulation amplitudes then they are said to be perfectly discriminated [15]. The implication of perfect discrimination is that the features of the two stimuli by which they are discriminated are detected by different labeled detectors. The thresholds (at the 75% correct level of performance on the fitted functions) will, of course, also be equal. Less than perfect discrimination between the two types of stimuli in the absence of a bias towards one or the other would be manifest in thresholds for the identification of each stimulus being higher than their respective thresholds for detection (because channels sensitive to different RF stimuli have been shown to be independent [10] and the reference stimulus, a circle, has no modulation and, therefore, represents a null point with respect to RF patterns, the results of these 2×2 forced choice experiments should be free of the criterion shift that can occur in such experiments [21]). A bias towards perception of one test stimulus type over the other will result in an excess of false positive identifications of that type, resulting in an apparent lowering of its threshold for identification at the expense of the identification threshold of the other type of pattern.

## Results

**Figure 2:**
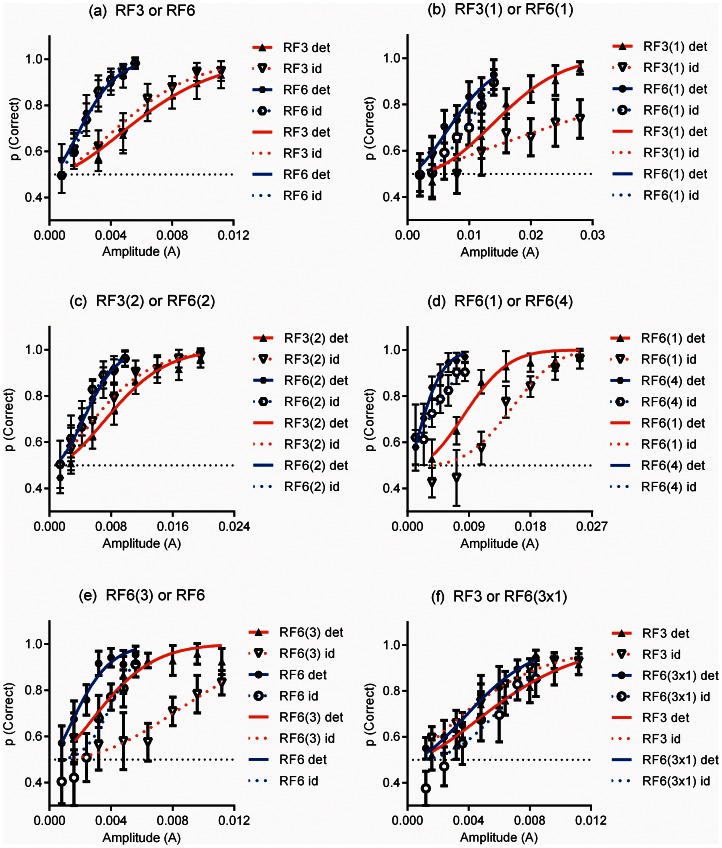
Psychometric functions describing performance of the group of observers. Probabilities of correct detection and identification performance of the group of observers are plotted against the amplitude of modulation, *A*, of the test stimulus. Error bars are 95% confidence intervals. The solid lines are Quick functions fitted to the detection data and dashed lines the identification data. The red lines denote performance pertaining to the first test stimulus of the pair and blue lines the second. If the solid and dashed lines are coincident for both stimuli the patterns are perfectly discriminated. The results of F tests to determine whether the fits described by the solid and dashed lines of each pair are significantly different statistically are presented in Table 1.

Thresholds for detection and identification of six pairs of stimuli were determined. Supra-threshold examples of the stimuli are shown in Figure 1 for comparison with the results shown in Figure 2.

Figure 1 shows examples of the pairs of patterns used in the experiment. Pair (a) comprises an RF3 and an RF6 pattern (A = 0.05 for all RF3 patterns and A = 0.0135 for RF6 patterns – these amplitudes are half those at which concave curvature features appear on the path and result in the deformation in examples of RF3 and RF6 patterns having approximately equal salience [22]). The patterns of Pair (b) have single cycles of RF3 and RF6 modulation. The patterns of Pair (c) have two cycles of modulation. Pair (d) comprises patterns with one and four cycles of modulation of the same frequency (RF6). The patterns of Pair (e) have the same frequency of modulation (RF6) but have three or six cycles of modulation. Finally, one pattern of Pair (f) is an RF3 pattern and the second has three cycles of RF6 modulation, which have a shorter wavelength but are distributed across alternate periods so that the points of maximum convex curvature in both patterns are, therefore, at intervals of 2π/3 radians.

Figure 2 shows psychometric functions fitted to the combined data of the four observers for the six pairs of stimuli. The data were combined in this way because they did not differ significantly across observers. The labelling, (a) to (f), of the panels corresponds to that used for the pairs of stimuli shown in Figure 1. The functions fitted to the detection data are illustrated by continuous lines and the functions fitted to the identification data by dashed lines. The fits are colour coded with red representing the data pertaining to the first of the stimuli of each pair (as presented in Figure 1) and blue the second. If the stimuli of the pair are perfectly discriminated the continuous and dashed lines of each colour will be coincident. Following Watson & Robson [15] we tested first whether the thresholds for detection and discrimination were statistically different. Extra sum of squares F tests were performed for each of the fits represented by the similarly coloured pairs of continuous and dashed lines. Data analysis was performed using GraphPad (GraphPad Prism version 5.00 for Windows, GraphPad Software, San Diego California USA, www.graphpad.com). Second we performed χ^2^ tests of the observers’ responses for each pair of stimuli following the statistical model of Watson & Robson [15]. The results of the F tests and χ^2^ tests are presented in Table 1.

**Table 1:** Statistical tests.

Stimulus Pair	First Stimulus of Pair	Second Stimulus of Pair	χ^ 2^ test
RF3 & RF6 (2a)	F = 3.587 (1,164), p = 0.0600	F = 0.5894 (1,164), p = 0.4437	χ^2^(27) = 5.7078, p>0.995
RF3(1) & RF6(1) (2b)	F = 502.0 (1,164), p<0.0001***	F = 15.07 (1,164), p<0.0001***	χ^2^(27) = 63.266, p<0.005
RF3(2) & RF6(2) (2c)	F = 7.701(1,164), p = 0.0062**	F = 0.4653 (1,164), p = 0.4961	χ^2^(27) = 8.3317, p>0.995
RF6(1) & RF6(4) (2d)	F = 74.64 (1,164), p<0.0001***	F = 12.40 (1,164), p = 0.0006***	χ^2^(27) = 68.086, p<0.005
RF6(3) & RF6 (2e)	F = 87.02 (1,164), p<0.0001***	F = 77.93 (1,164), p<0.0001***	χ^2^(27) = 166.40, p<0.005
RF3 & RF6(3x1) (2f)	F = 14.57 (1,164), p = 0.0002***	F = 22.82 (1,164), p<0.0001***	χ^2^(27) = 44.140, p<0.05

Extra sum of squares F tests were used to determine if the fits to the data describing thresholds for detection and identification of the patterns of each test Pairs 1(a)-(f) were statistically different for the group of observers (see Figure 2). The thresholds (at 75% correct performance) are assumed to be the same unless p<0.05. Three asterisks highlight p<0.001, two p<0.01 and one p<0.05. χ^2^ tests were performed as per Watson & Robson [15] to determine if the patterns were perfectly discriminated. Adopting the criteria that p>0.95 indicates perfect discrimination and p<0.05 not the tests show that only Pairs 1(a) and 1(c) are can be considered perfectly discriminated and the other pairs are demonstrably not perfectly discriminated.

**Figure 3:**
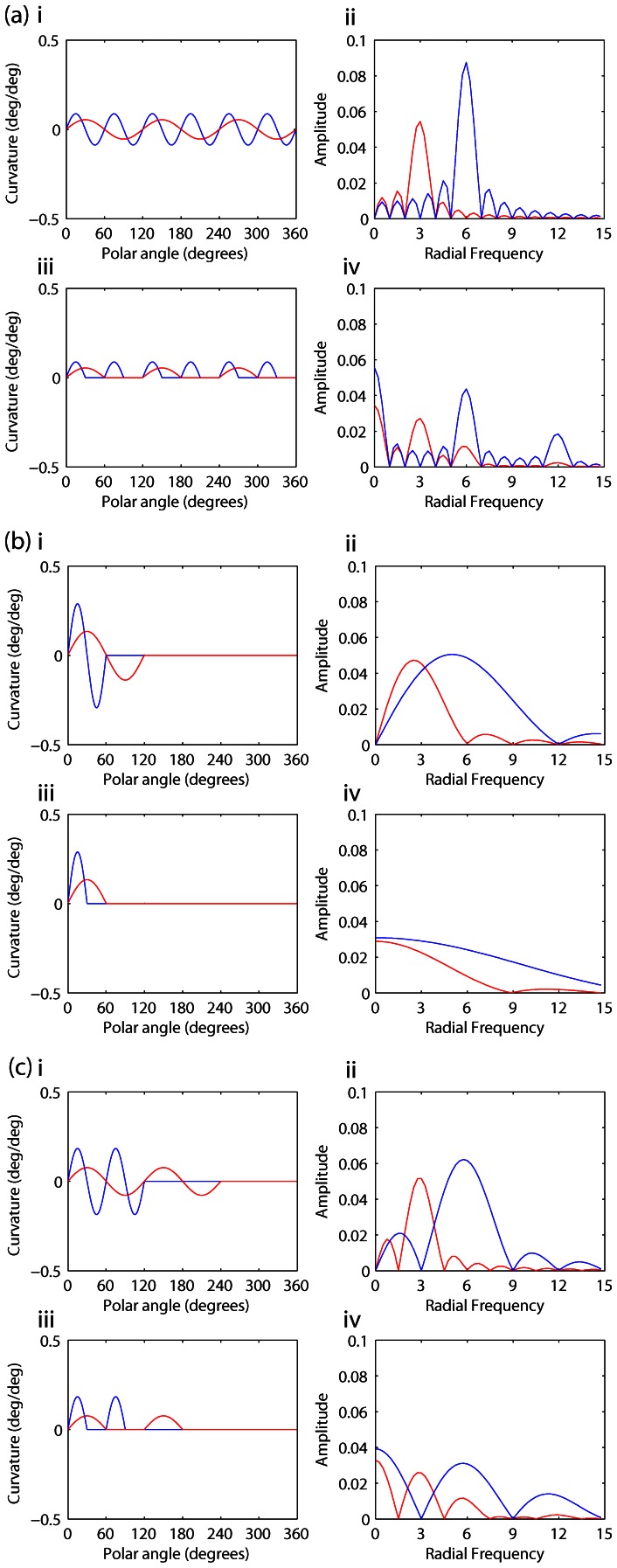
Curvature and curvature frequency spectra for Pair 1(a), 1(b) and 1(c). The graph (a)i plots the curvature (rate of change of orientation with respect to circular) of the complete RF3 (red) and RF6 (blue) patterns (Pair 1(a)) as a function of the polar angle. The graph (a)iii plots the rectified curvature. The curvature shown is that derived for the amplitudes of modulation at the threshold for detection of the patterns. When the curvature is tighter than a circular arc at the base radius R_0_ the curvature is positive and when it is shallower it is negative. The graphs (a)ii and (a)iv in the right hand column are the frequency spectra of the curvature measures shown in (a)i and (a)iii respectively. In (b) modulation of curvature is constrained to a single cycle (for the rectified measure of curvature it is constrained to a half cycle). Data pertaining to the single cycle RF3 pattern is in red and RF6 in blue. For the un-rectified measure of curvature, (b)i, distinct peaks distinguishing the RF3 and RF6 modulation can be discerned in the frequency spectra, (b)ii. For the rectified measure, however, this is not so. In (c), data pertaining to Pair 1(c), modulation of curvature is constrained to two cycles (RF3 modulation in red and RF6 in blue). Distinct peaks distinguishing the RF3 and RF6 patterns can be seen across the frequency spectra for both the un-rectified and rectified curvature measures.

As discussed in the introduction, Poirier and Wilson suggest that the function describing curvature (where curvature is defined as the rate of change of orientation with respect to circular) versus polar angle is rectified prior to shape analysis. Poirier and Wilson propose that the shape representations might be approximated by sinusoids and so the correlation can be implemented as a Fourier transform. Therefore, for comparison with the results Figure 3 displays the curvature, at threshold for detection, of the paths describing the patterns of stimulus Pairs (a), (b) and (c) as a function of polar angle, and also the frequency spectra of these functions. The curvature measure (and spectrum) displayed in red is that pertaining to the first stimulus of each pair (RF3 or truncated RF3) and that displayed in blue pertains to the second (RF6 or truncated RF6). The curvature measure used is the rate of change of orientation with respect to circular where the orientation is given by Eq. (2). This is the measure of curvature used by Poirier and Wilson [9] although their model subsequently half wave rectifies the curvature such that only curvature that is convex with respect to circular is used in the shape analysis. Curvature measures are displayed in the graphs on the left (i = unrectified, iii = rectified). The frequency spectra for the curvature measures are shown in the graphs of the right (ii = unrectified, iv = rectified).

**Figure 4:**
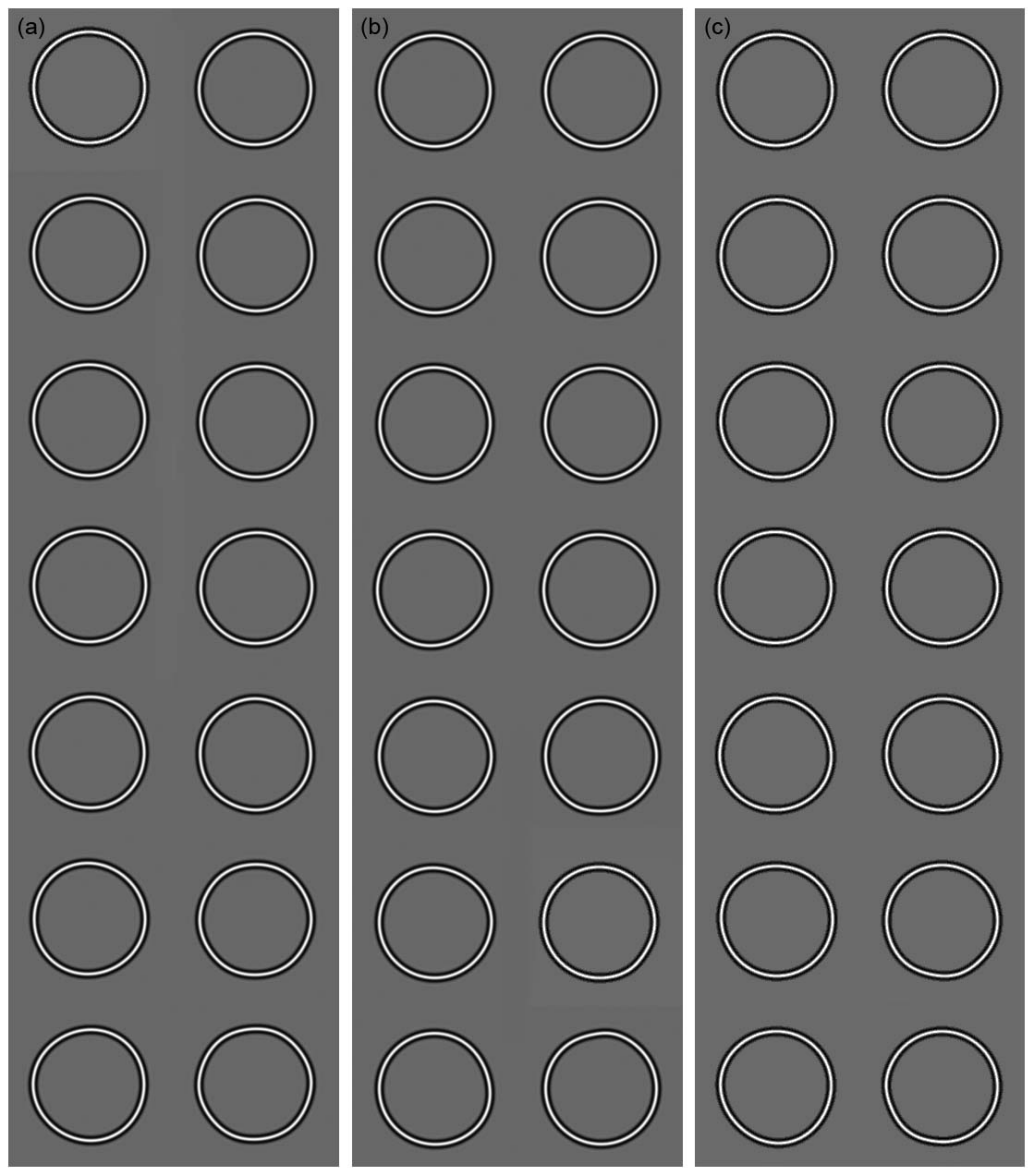
Example stimuli for the conditions represented by Pair 1(a), 1(b) and 1(c). In (a), the RF3 patterns are shown in the left hand and RF6 patterns in the right hand column. Amplitude of modulation increases from top to bottom. The amplitudes of modulation used in the stimuli are those used in the experiment for the method of constant stimuli. It is evident from these examples that when the modulation can be detected the patterns can be discriminated. Example stimuli for the condition represented by Pair (b) are shown in (b). The single cycle RF3 patterns are shown in the left hand and single cycle RF6 patterns in the right hand column. The patterns are in random phase. Again the amplitudes shown are those used in the experiment. It can be seen from these examples that modulation can be detected before it is of sufficient amplitude to allow discrimination. In (c) example stimuli for the condition represented by Pair (c) are shown. Two cycle RF3 patterns are shown in the left hand and two cycle RF6 patterns in the right hand column. The figure illustrates that when two cycles of modulation are present in the stimuli the RF3 and RF6 patterns can be identified at their thresholds for detection.

Figure 4 shows examples of the stimuli, for Pairs (a), (b) and (c), with the amplitudes of modulation at which they were displayed in the experiment. The seven stimuli top to bottom of the figure represent the increasing amplitudes of the stimuli used in the method of constant stimuli.

Figure 2 (a) shows that fits to detection (solid lines) and identification data (dashed lines) are near identical (and not significantly different) for each complete RF pattern. This demonstrates that, at their thresholds for detection, RF3 and RF6 patterns can be discriminated. The curvature and frequency spectra for these conditions are shown in Figure 3(a). Figure 3(a)i shows continuous measures of curvature around the patterns for the RF3 (red) and RF6 (blue) patterns and Figure 3(a)ii their frequency spectra (similarly colour coded red for the RF3 pattern and blue for the RF6). Figure 3(a)iii shows the same measures of curvature half wave rectified and Figure 3(a)iv the frequency spectra of these rectified curvature measures. Discrete peaks are evident at 3 and 6 cycles per degree for the RF3 and RF6 patterns respectively. This is true for both the non-rectified and rectified curvature measures. Example stimuli for this stimulus pair are shown in Figure 4(a). The RF3 stimuli are shown in the left hand and the RF6 in the right hand column. The stimuli are in random phase and increase in amplitude from top to bottom. The amplitudes used match those used in the experiment for the method of constant stimuli. It is evident from these examples that when the amplitude of modulation is visible the patterns can be discriminated.

For Pair 1(b) when only a single cycle of modulation is present, Figure 2(b) shows that identification thresholds are significantly higher than detection thresholds, that is, the patterns are not perfectly discriminated. This result indicates that the curvature information over a single cycle of modulation is inadequate to permit identification at the threshold for detection (see also Bell, Dickinson and Badcock [23] and Wang and Hess [24]). Graphs of the curvature versus polar angle for this condition, and the frequency spectra are shown in Figure 3(b). For these stimuli, where modulation is restricted to a single cycle, there is a large qualitative difference between the frequency spectra of the un-rectified (i) and rectified (iii) curvature measures. For the rectified curvature measures the frequency spectra peak at a frequency of zero, whilst for un-rectified curvature measures the spectra peak close to three and six cycles per 2π radians. While there is substantial overlap of the peaks of the spectra for the un-rectified curvature measures of the two patterns, the amplitude of the frequency spectrum for the single cycle RF3 pattern is zero at a frequency of six cycles per 2π radians, providing a potential discriminator of the patterns. The patterns might be reliably discriminated at their threshold for detection on the basis of the response or otherwise of a labeled detector for a frequency of six cycles per 2π radians in a continuous measure of curvature around the pattern. That these patterns are not discriminable at their thresholds for detection suggests that the analysis of periodicity is most likely to have been restricted to the points of convex curvature extrema, or corners. Figure 4(b) shows examples of stimuli used for this condition of the experiment. It is evident from the figure that modulation within the patterns can be detected at amplitudes that do not allow the patterns to be discriminated.

Figure 2(c) shows that when two cycles of modulation of different frequency (or period) are present in both patterns, the patterns can be discriminated at their detection thresholds, indicating that repetition of a curvature feature is sufficient for discrimination as long as the period of repetition in the patterns is sufficiently different. Figure 3(c) shows the curvature measures, un-rectified, i, and rectified, iii, and curvature frequency spectra for the patterns with two cycles of modulation (Pair 1(c)). Discrete peaks in the frequency spectra for both the un-rectified and rectified curvature measures are evident. Figure 4(c) presents example stimuli for the condition of the experiment testing the discriminability of the stimuli of Pair 1(c), RF3 and RF6 patterns with 2 cycles of modulation. In common with the complete RF patterns of Pair 1(a) the patterns can be discriminated as soon as the modulation can be detected.

The results shown in Figure 2(d) (one or four cycles of an RF6) and 2(e) (three or six cycles of an RF6) demonstrate that, when the patterns have the same modulation frequency (period of modulation), they cannot be discriminated, at threshold, on the basis of the number of cycles of modulation present. This finding is consistent with a periodicity argument, but also demonstrates that identification at threshold is independent of any feature counting strategy, which would predict the patterns to be discriminable based on number of cycles. Finally, the results shown in Figure 2(f) demonstrate a propensity for the observers to over-report the RF3 stimulus as presented and under-report the stimulus with the three non-consecutive cycles of RF6 modulation. This can be seen by the opposite horizontal shifts between detection (solid) and identification (dashed) lines in 2(f) for red (RF3) and blue (RF6 3x1). Here the periodicity, or angular separation between convex curvature features on both patterns is consistent with an RF3 and so, the over-reporting of RF3 is consistent with the angular separation between corners being a primary cue for representing shape; i.e. a periodicity argument.

Within Table 1 one result can be considered anomalous. The threshold for identification of the two cycle RF3 pattern is slightly lower than the threshold for detection and this difference is statistically significant. This is suggestive of an excess of false positive identifications. However, because the converse is not true of the RF6 pattern with two cycles, which is shown to have the same probabilities for identification and detection, this result is most likely a statistical aberration. The χ^2^ test indicates that these patterns are perfectly discriminated.

## Discussion

In this study we have shown that RF3 and RF6 patterns are perfectly discriminated. That is to say that the probability of correct identification of the test stimulus, at any particular modulation amplitude, is equal to the probability of its detection. Measures of the feature of the patterns upon which discrimination is based must, therefore, be encoded in discrete detectors. In the 2x2FC test only one of the two possible stimuli is presented in any trial and so these detectors must be labeled as corresponding to particular magnitudes of that feature [15], frequency (of modulation or period of repetition of a curvature feature) in this case. We can say, therefore, that mechanisms responsible for the detection of RF3 and RF6 patterns near threshold are non-overlapping on this critical signal dimension. This result is consistent with the sub-threshold summation results of Bell and Badcock [10]. Patterns with single cycles of RF3 and RF6 modulation, however, are not perfectly discriminated. This result rules out the possibility that discrimination of the patterns might depend upon the detection of discrete values of curvature, or a measure of the subtended angle between the point of maximum curvature and the point of minimum curvature in a single modulation cycle. RF3 and RF6 patterns with two cycles of modulation are perfectly discriminated despite the fact that the number of points of maximum convex curvature is the same in each pattern. This demonstrates the importance of the measure of periodicity. That the periodicity inherent in a single cycle is not used suggests that the analysis of the form of RF patterns exploits the periodicity of curvature maxima for these stimuli. Such analysis might be made after an explicit half wave rectification of curvature with respect to circular, as argued by Poirier and Wilson [9] but this cannot be confirmed by the current data. These experiments cannot, therefore, conclude that curvature features that are concave with respect to the centre of the pattern are unimportant in the analysis of shape primitives. The frequency spectrum of a continuous measure of curvature would, however, still be peaked near the frequency modulation, despite the d.c. offset in curvature, even for patterns with a single cycle of modulation. We can, therefore, say with some confidence that the analysis of shape does not proceed by a decomposition into radial frequency components via cross correlation of a continuous measure of curvature with sinusoids at a range of frequencies.

Having shown that patterns of the same frequency but which differ in number of cycles cannot be discriminated at threshold, we can also rule out the possibility that pattern identification at threshold is dependent upon differing numbers of curvature features. The element of shape that best accounts for our data is the angle subtended at the centre of the pattern by adjacent corners. Repetition of this feature results in a lowering of threshold but patterns with different numbers of repetition cannot be discriminated at threshold. Therefore we conclude that it is the periodicity of corners which identifies the patterns used in this study.

Attneave [1] and Feldman & Singh [3] have shown that the information content in an image is concentrated at the points of maximum curvature of boundaries. The models of Poirier and Wilson [9] and Pasupathy and Connor [7], [8] recognize the importance of such points and relate their positions to the centre of objects. Shapes in these models are represented by the angular distribution of curvature features around the pattern centre, relative to a reference direction in polar space. If curvature is defined as the rate of change of orientation with respect to circular, as it is by Poirier and Wilson, then this form of representation is indifferent to distance from the centre to each curvature feature, providing a size invariant representation of shape. A recent paper argues the response of neurons of V4 are tuned for acute convex or concave curvature [25]. The thesis of the paper is that by strongly weighting acute curvature a sparse coding is achieved at this level of representation. Such a representation would lend itself to a representation of shape based on the polar angle between such curvature features.

This representation is consistent with the Poirier and Wilson model, the Pasupathy and Connor model and previous work that has shown that shape in RF patterns is encoded by the polar angle separation of curvature features at the centre of the pattern rather than absolute distance measured as visual angle [23], providing support for a size invariant representation, an important constraint imposed by established properties of the visual system [26].

The results of the current study extend the previous work by explicitly demonstrating that the discrimination of shapes is similarly based on the periodicity of corners. Previous research and modeling has suggested that the angular separation of features could be used to identify shapes but had provided no evidence in support of this claim. It was necessary to use near threshold modulations to demonstrate this result and even with such weakly modulated shapes independent processes can be revealed. It is, of course, necessary to now determine that the results do extend to supra-threshold shape changes. The patterns used here had their phases (shape orientations) randomized, thereby preventing the observers attending only to a part of the pattern which was known to contain a specific curvature feature. This also prevents observers from referencing a feature to a particular direction from the pattern centre and so the experiment does not test whether shape representations exist that are labelled for orientation. It can, however, be concluded that patterns which are randomized in phase are perfectly discriminated when they contain adjacent corners which subtend different angles at the pattern centre. In this sense the pattern representation revealed is invariant to orientation in the fronto-parallel plane. Such a representation has the capacity to generalize to all simple closed contours. If complex shapes are decomposed at matched points of concavity as proposed by Hoffman & Richards [27] and Biederman [28] this representation could provide the basis for the identification of the primitives of all shapes. Pasupathy and Connor examined the response of V4 neurons to shapes defined by the polar positions of curvature features around the centre of each shape (for a review see Connor [29]). In contrast to Poirier and Wilson they considered curvature features which were both convex and concave with respect to the centre of the pattern and found neurons that were selective for curvature features which were concave with respect to the centre of the pattern. The individual V4 neurons were shown to be selective for particular curvature features in particular polar directions relative to the centre of the pattern and similar selectivity for the polar position of curvature features has been observed higher in the visual processing hierarchy [30], [31]. This observation seems to imply that the shape is represented relative to a reference direction in the fronto-parallel plane but, as has been previously stated, this type of representation does not lend itself to recognition of the same object which has undergone different rotational transformations. However the result might simply reflect the retinotopic arrangement of the neural machinery, with the curvature feature detectors having a particular spatial relationship to the neurons that integrate series’ of such features with reference to a pattern centre. In our study the phase of the patterns was randomized and therefore insensitive to the absolute polar position of particular curvature features but it is clear that different patterns were perfectly discriminated irrespective of phase differences. Our results imply that an orientation invariant representation of shape might be encoded as a frequency spectrum which represents the periods between successive corners on the boundary of an object. This representation could be subordinate to the representations proposed by Pasupathy and Connor [8] and Poirier and Wilson [9] for supra-threshold stimuli but may provide a size and orientation invariant representation of shape for impoverished stimuli or stimuli encountered under challenging circumstances.
